# Combined endoscopic stenting and laparoscopic stent fixation for benign gastric tube stricture after esophagectomy: a case report

**DOI:** 10.1186/s40792-023-01787-5

**Published:** 2023-11-30

**Authors:** Koji Shindo, Kenoki Ohuchida, Tomohiro Nagasue, Taiki Moriyama, Fumika Goto, Koji Tamura, Kinuko Nagayoshi, Yusuke Mizuuchi, Naoki Ikenaga, Kohei Nakata, Masafumi Nakamura

**Affiliations:** 1 Department of Surgery and Oncology, Kyushu University, 3-1-1 Maidashi, Higashi-ku, Fukuoka City, Fukuoka 812-8582 Japan; 2 Department of Medicine and Clinical Science, Kyushu University, 3-1-1 Maidashi, Higashi-ku, Fukuoka City, Fukuoka 812-8582 Japan

**Keywords:** Laparoscopic stent fixation, Stent migration, Benign stricture, Gastric tube reconstruction, Video-assisted subtotal esophagectomy

## Abstract

**Background:**

There are several options for the treatment of gastrointestinal stricture, including endoscopic stent placement and bypass surgery. However, a benign stricture is difficult to manage in a reconstructed gastric tube in the thoracic cavity owing to the technical difficulty of bypass surgery, and the possibility of stent migration.

**Case presentation:**

A 78-year-old woman was admitted to our hospital for treatment for her inability to eat. She had undergone video-assisted subtotal esophagectomy with retromediastinal gastric tube reconstruction 7 years earlier. At the current admission, there was a severely dilated gastric tube in the thoracic cavity with a soft stricture immediately anterior to the spine. Conservative therapy was ineffective; therefore, endoscopic stenting was performed. However, the stent migrated to the upper side of the stricture because the stricture was mild, and the stent was not fixed in the gastric tube. Next, endoscopic stent placement followed by laparoscopic stent fixation was performed. The stent was patent and worked well, and the patient’s body weight increased. However, the stent collapsed 2 years later, with recurrence of symptoms. Stent-in-stent placement with an over-the-scope clip was performed, and the second stent was also patent and worked well.

**Conclusions:**

Laparoscopic stent fixation with endoscopic stent placement could be an effective option for patients with a benign stricture in the reconstructed gastric tube.

## Background

There are several options for the treatment of gastrointestinal stricture, including endoscopic stent placement and bypass surgery. The estimated rate of malignant bowel obstruction is 2% of all patients with advanced malignancy [1], and patient management to maintain quality of life has been described [2–4]. However, it is difficult to manage or treat a benign stricture because the goal is to maintain stricture patency for prolonged periods. Occasionally, a benign stricture is mild, and a stent cannot fix well in the stricture, which leads to stent migration. Bypass surgery is also a management option for benign stricture. However, this method is not feasible in patients with poor performance status or when bypass surgery is technically difficult.

Herein, we report a case of a benign stricture in the gastric tube after esophagectomy, which was treated by combined endoscopic stent placement and laparoscopic fixation.

## Case presentation

**Fig. 1:**
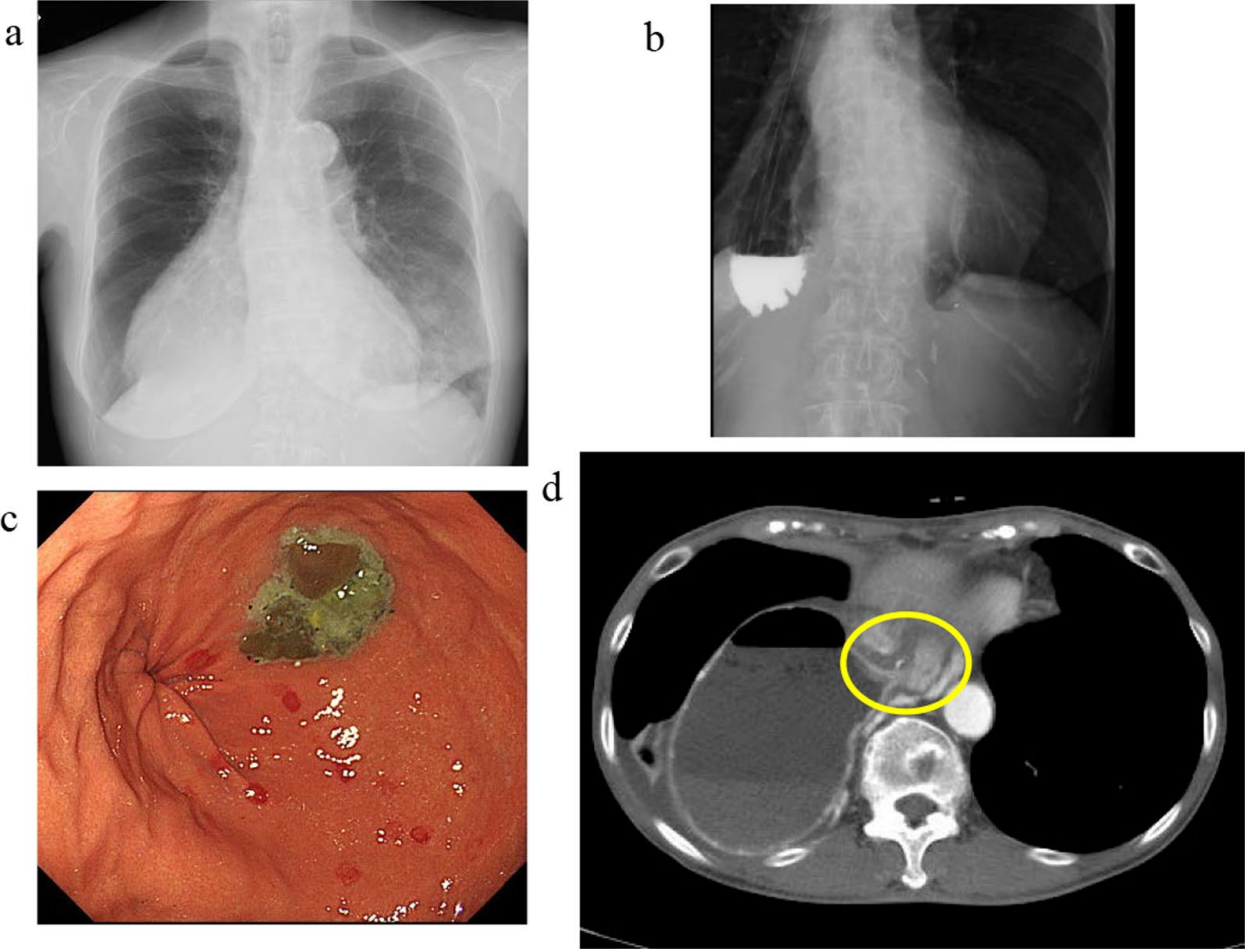
Examination findings when the patient complained of vomiting. **a** A dilated gastric tube is visible in the thoracic cavity on chest X-ray. **b** Contrast medium is retained in the thoracic part of the gastric tube on fluoroscopy. **c** On upper gastrointestinal endoscopy, deflection and torsion of the gastric tube is visible at the site of the thoracoabdominal transition. **d** On CT, a severely dilated gastric tube is visible in the thoracic cavity with a stricture (yellow circle). *CT* computed tomography

**Fig. 2:**
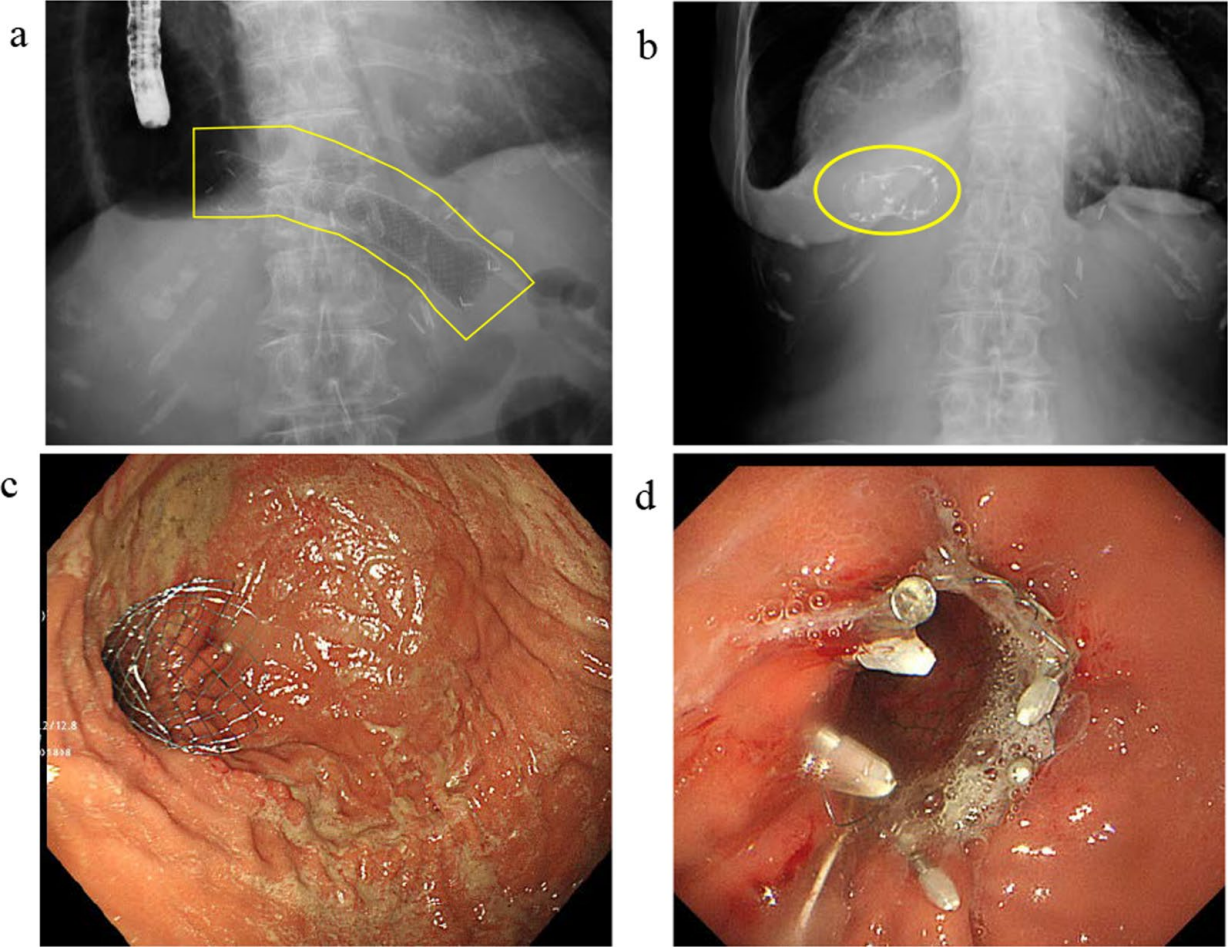
Endoscopic stent placement. **a** A covered stent (area outlined in yellow) was placed safely, without complications. **b** The stent (yellow circle) migrated to the upper side of the stricture several days later. **c** and **d** A bare metal stent was placed with clipping 1 day before surgery

**Fig. 3:**
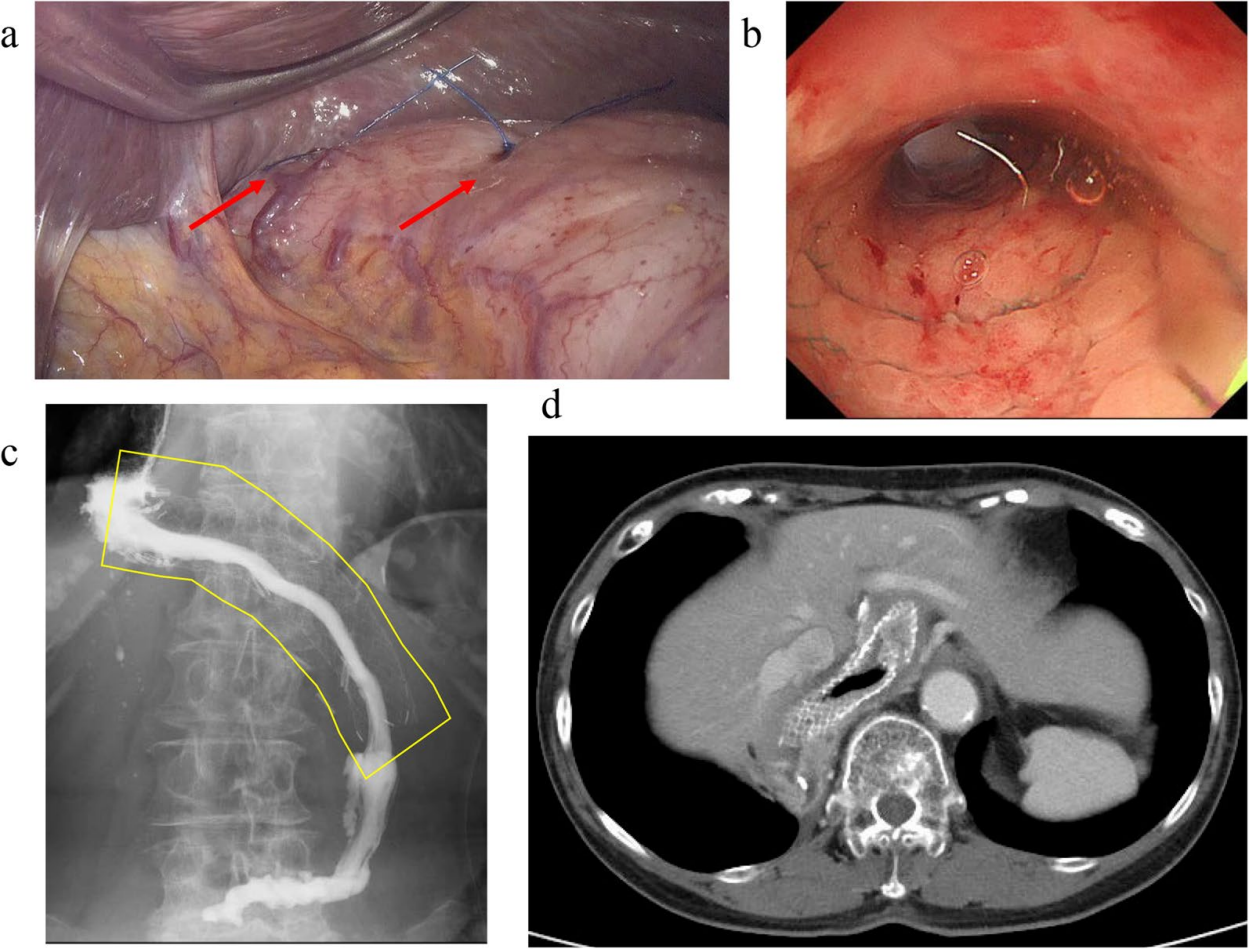
Findings during laparoscopic stent fixation (LSF). **a** We fixed the stent through the full-thickness gastric wall with 3-0 Prolene at the oral side of the antrum and lower gastric body (red arrows). **b** Needle placement through the stomach wall was confirmed by endoscopy. **c** The stent (area outlined in yellow) was patent after surgery, and contrast medium passed through easily. **d** Postoperative CT showing no dilated gastric tube in the thoracic cavity. *CT* computed tomography

**Fig. 4:**
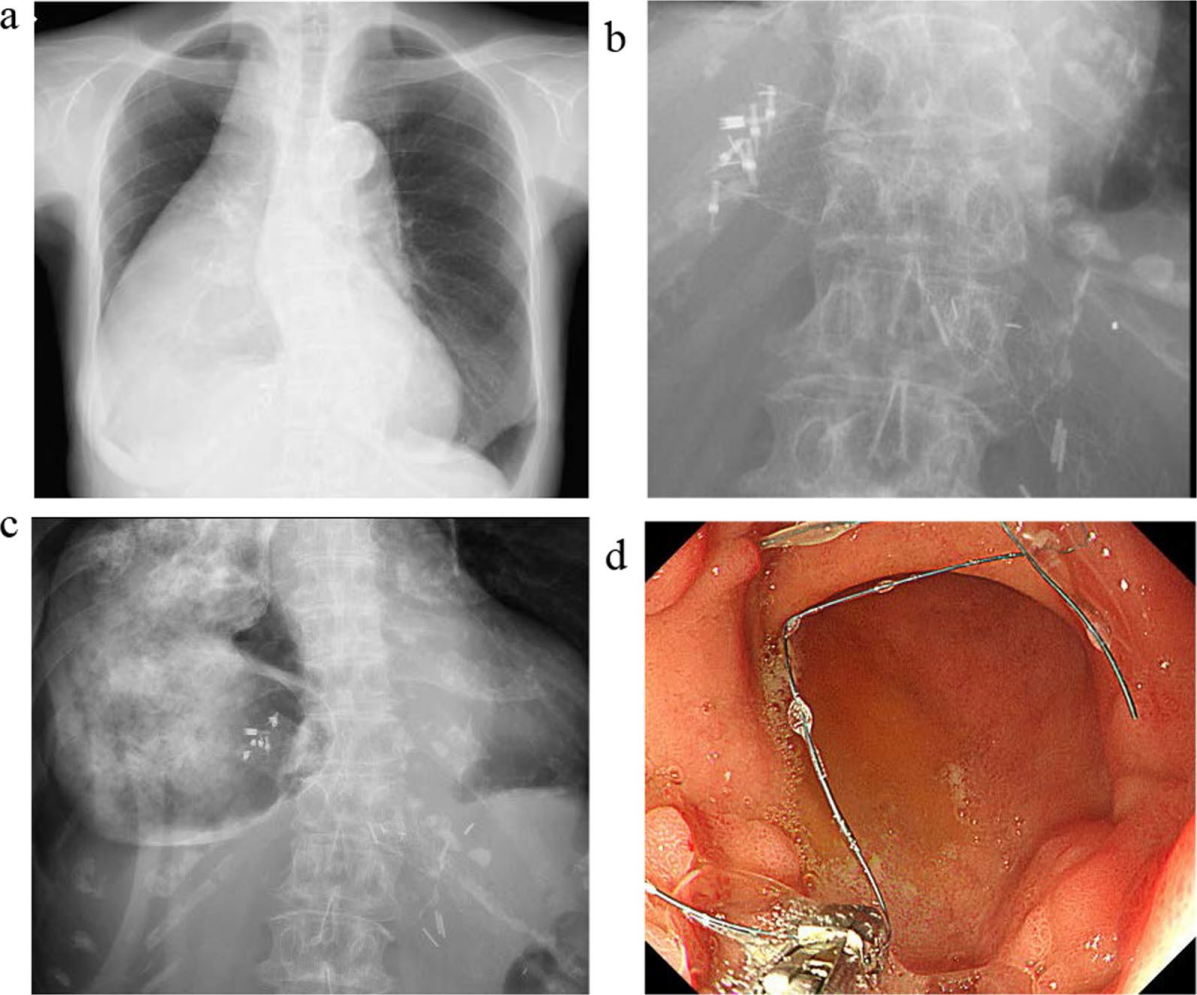
Examination findings when the patient complained of vomiting recurrence 2 years postoperatively. **a** A dilated gastric tube is visible in the thoracic cavity on chest X-ray. **b** The previously placed stent was well-fixed but had collapsed distally. **c** Contrast medium is retained in the thoracic part of the gastric tube on fluoroscopy. **d** The distally collapsed stent is visible on endoscopy

**Fig. 5:**
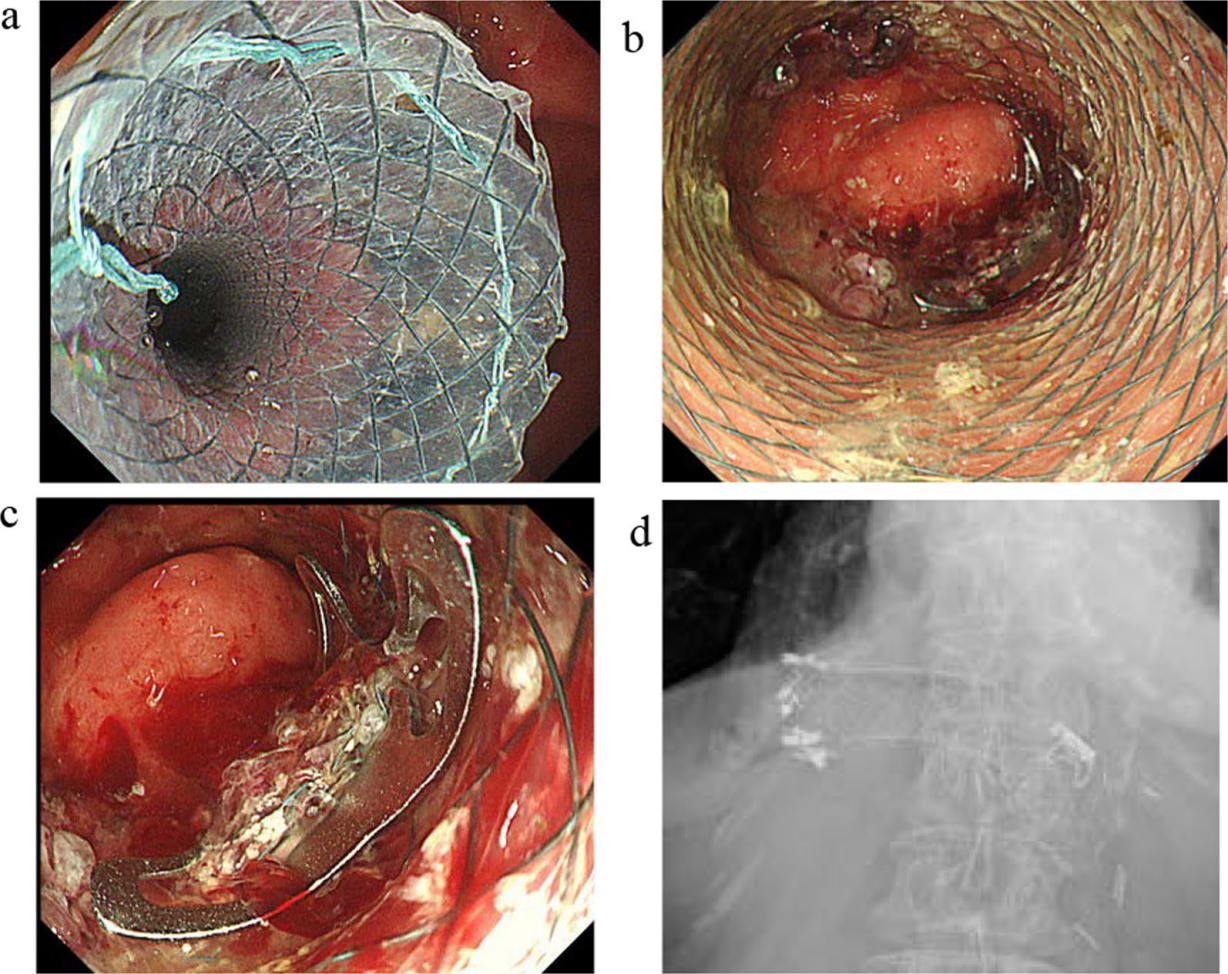
Endoscopic stent-in-stent placement. **a** A stent was placed inside the previous stent. **b** Patency of the new stent was confirmed. **c** The stent was fixed with the previous stent using OTSCs. **d** On X-ray, the new stent is patent. *OTSC* over-the-scope clip

**Fig. 6:**
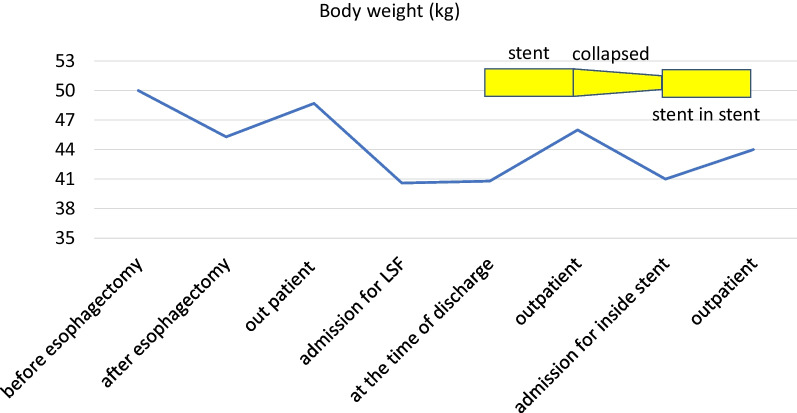
Changes in the patient’s body weight over time

A 78-year-old woman was admitted to our hospital for treatment for her inability to eat. She had undergone neoadjuvant chemotherapy and video-assisted subtotal esophagectomy with retromediastinal gastric tube reconstruction 7 years earlier. She had also undergone partial resection of her left breast with axillary lymph node dissection for breast cancer, and radiation with adjuvant hormone therapy 3 years earlier. She complained of vomiting and a 6-kg loss in body weight over 1 year. On physical examination, there were no abnormal findings other than emaciation. On chest X-ray, there was a dilated gastric tube in the thoracic cavity (Fig. 1a). Decompression using a nasogastric tube was performed but was ineffective. On fluoroscopy after decompression, the contrast medium remained in the thoracic part of the gastric tube and barely passed through to the abdominal part (Fig. 1b). On upper gastrointestinal endoscopy, there was no severe stricture; however, deflection and torsion at the site of the thoracoabdominal transition was observed (Fig. 1c). For several years after the esophagectomy, computed tomography had revealed no abnormal findings. However, with the onset of the current complaint of inability to eat, CT revealed a severely dilated gastric tube in the thoracic cavity with a stricture immediately anterior to the spine (Fig. 1d), with no evidence of cancer recurrence or dissemination. The stricture was soft, and the endoscope could pass through easily. Bypass surgery was not feasible because the stricture was in the thoracic cavity. We performed stent placement using a covered through-the-scope esophageal stent with clipping, in accordance with previous reports (Fig. 2a) [5, 6]. However, the stent migrated to the upper side of the stricture several days after the patient restarted oral intake (Fig. 2b). Next, we planned to perform stent fixation using laparoscopy. We performed endoscopic stent placement using a bare metal stent (pyloric duodenal D-type stent) (Fig. 2c) with clipping (Fig. 2d) 1 day before surgery, with laparoscopic stent fixation (LSF) the next day. Intraoperatively, we explored the abdominal cavity and found no evidence of dissemination or compression of the gastric tube. We fixed the stent to the full-thickness gastric wall with 3-0 Prolene (Ethicon Inc., Somerville, NJ, USA) on the oral side of the antrum and lower gastric body (uppermost part in the abdominal cavity) (Fig. 3a, red arrow) and confirmed needle placement using endoscopy (Fig. 3b). Jejunal tube insertion was added to address possible difficulty with the passage of gastrointestinal contents. Postoperatively, the stent was fully patent and did not migrate. Contrast medium passed freely through the stent (Fig. 3c), and there was no evidence of dilation of the gastric tube in the thoracic cavity on CT (Fig. 3d). The patient’s oral intake and body weight improved markedly after surgery. However, she was readmitted after recurrence of symptoms, namely vomiting and inability to eat, 2 years after the operation. X-ray (Fig. 4a and b), fluoroscopy (Fig. 4c), and endoscopy (Fig. 4d) showed that the stent had collapsed, with recurrence of a stricture and gastric dilation in the thoracic cavity. Therefore, we performed endoscopic stent-in-stent placement (Fig. 5a and b) using over-the-scope clip fixation (Fig. 5c) to the previous stent. We chose a covered esophageal stent to achieve long-term patency. The stent-in-stent procedure was successful, and safe placement and patency were confirmed on X-ray (Fig. 5d). After this procedure, the patient’s oral intake and body weight improved again (Fig. 6). Unfortunately, recurrent breast cancer (invasive lobular breast cancer) occurred 4 months after this procedure, and palliative care was initiated.

## Discussion

In operations for gastrointestinal cancer, laparoscopic surgery and robot-assisted surgery are widely accepted in Japan [7]. Generally, we performed thoracoscopic esophagectomy in the prone position and retromediastinal gastric reconstruction for esophageal cancer before robotic surgery was covered by national insurance in Japan. Despite this minimally invasive surgery, half of the patients who underwent esophagectomy experienced postoperative functional disorders, such as delayed gastric emptying (DGE), dumping syndrome, and reflux with dysphagia [8], especially those who developed postoperative complications [9]. Jezerskyte et al. reported that a decrease in short- or long-term quality of life after esophagectomy is unrelated to postoperative complications but to the nature of esophagectomy and reconstructive procedures [10]. Various interventions have been performed to resolve these issues; however, the ideal solution is still unclear.

The most important factors in achieving improvement in patients’ postoperative conditions are the route of the reconstructed gastric tube and the size of the preserved stomach. We used sub-whole stomach with retromediastinal reconstruction, and we retract the stomach to the abdominal side and fix the stomach to the diaphragm around the hiatus after anastomosis to avoid having the stomach drawn into the thoracic cavity by negative pressure. Regarding the size of the reconstructed gastric tube, thoracic stomach syndrome can occur and cause chest discomfort after eating following esophagectomy with whole-stomach reconstruction [11–13]. Additionally, the causes of DGE vary and include bilateral vagotomy and pylorus paralysis due to automatic nerve imbalance. Furthermore, it is difficult to treat a soft stricture at the upper side of the pylorus, as in our case. We have rarely experienced deflection and torsion of a sub-whole stomach in the thoracic cavity leading to obstruction of the passage of food. Zhan et al. reported that a narrow gastric tube has a slight advantage regarding the avoidance of DGE compared with whole stomach in reconstruction [11]. We chose sub-whole stomach with retromediastinal route in reconstruction previously, but we have changed the reconstruction route from retromediastinal to retrosternal, and we use a narrow gastric tube.

Erythromycin (motilin receptor agonist) as a treatment for DGE after esophagectomy is effective [14]; however, this treatment is unsuitable for long-term use. If the cause of DGE is pylorospasm, endoscopic dilatation is effective. Additionally, gastrojejunal bypass may resolve this issue, but when the site of the stricture is in the thoracic cavity, as in our case, the bypass approach is not feasible. Re-operation such as removal of gastric tube with re-reconstruction was too invasive for benign stricture, and we considered to perform partial gastrectomy to make gastric tube straight in thoracic cavity. However, since the dilated area was the greater curvature side, we decided not to perform thoracic surgery to avoid injury of feeding artery of gastric tube. Our patient had deflection of the gastric tube into the thoracic cavity. However, she was able to eat for 7 years without symptoms. Therefore, we considered that the cause of the benign stricture was a change in the shape of the gastric tube over time with decreased peristaltic activity due to aging. Initially, we attempted decompression using a nasogastric tube and medication for 2 weeks to increase peristalsis; however, these measures were ineffective. We then attempted to correct the shape of the gastric tube using an ileus tube, but this procedure also failed. In this case, we tried medication of acotiamide hydrochloride hydrate, metoclopramide, and some other Japanese herbal Kampo medicines including ghrelin potentiator Rikkunshito using nasogastric tube or ileus tube through the stricture for over two weeks, but they were not effective. Regarding the loss of peristalsis in the gastrointestinal tract, peristalsis is also present in the gastric tube; therefore, age-related decrease in peristaltic activity appears likely, in our case [14, 15]. In such cases, retrievable metallic stent [16] is not suitable as they do not address the cause of the weakened peristalsis, and no improvement is expected after the stent removal. Generally, higher numbers of older patients are undergoing surgery compared with previous years; therefore, a similar situation to that in our patient could become more frequent in the near future.

Stent placement is considered a promising approach for strictures in the gastrointestinal tract. It is the most common modality performed worldwide to obtain lifelong relief of dysphagia [17]. However, there is no long-term evidence regarding stent efficacy for benign strictures; therefore, temporary stenting using a covered stent with removal after improvement is ideal [5, 6]. According to the European Society of Gastrointestinal Endoscopy guidelines, temporary placement of self-expandable stents for refractory benign esophageal strictures is weakly recommended [18]. However, Dan et al. reported that removable esophageal stents have poor efficacy in the treatment of benign esophageal strictures owing to the high rate of migration (53%) and subsequent chest pain [19]. In our case, the stricture was not severe; therefore, stent fixation after placement was very difficult. Moreover, stent migration occurs in approximately 28.6% of patients, even in those with malignancy [16]. In fact, in our patient, the stent migrated to the upper side of the gastric tube before we considered a surgical approach.

As a solution, we performed LSF as a less invasive approach compared with thoracoscopic bypass surgery. Over-the-scope clip (OTSC) [20], or endoscopic suturing [21] could be feasible for stent fixation endoscopically. However, when the stricture develops in the sub-whole gastric tube, there are few places where the stent can securely hit to the wall. Regarding endoscopic suturing, the gastric tube in retromediastinal route is surrounded by lung, aorta, pericardial sac, and liver, then it is not absolutely safe. In such gastric tube cases, we believe this LSF is better approach to fix the stent than OTSC or endoscopic suturing because we can choose the point to fix freely and safely. With retromediastinal gastric tube reconstruction, fistulae may develop between the gastric tube and aorta or the membranous part of the trachea, as adverse events [5]. Full informed consent from the patient and scheduled imaging follow-up is necessary with this type of adverse event.

## Conclusions

Recently, the number of esophagectomies in elderly patients has been increasing, as has long-term survival. In such cases, benign strictures of the reconstructed gastric tube may occur. LSF with endoscopic stent placement could be an effective treatment option.

## Data Availability

Not applicable.

## Abbreviations

CT: Computed tomography
LSF: Laparoscopic stent fixation
DGE: Delayed gastric emptying
